# Bronchial artery hypertrophy-associated perioperative pulmonary hemorrhage in cardiovascular surgery: a case report

**DOI:** 10.1186/s40792-022-01432-7

**Published:** 2022-04-29

**Authors:** Shinji Abe, Yasuhiro Kamikubo, Nobuyasu Kato, Hiroki Kato, Tomonori Ooka, Yasushige Shingu, Satoru Wakasa

**Affiliations:** 1 Department of Cardiovascular and Thoracic Surgery, Faculty of Medicine and Graduate School of Medicine, Hokkaido University, Kita 15, Nishi 7, Kitaku, Sapporo 060-8638 Japan; 2 Department of Cardiovascular Surgery, Kushiro-City General Hospital, Kushiro, Japan

**Keywords:** Bronchial artery hypertrophy, Pulmonary hemorrhage, Embolization

## Abstract

**Background:**

Pulmonary hemorrhage is a life-threatening complication of cardiovascular surgery. Bronchial artery hypertrophy, a rare pathology associated with inflammatory and ischemic respiratory diseases, increases the risk of pulmonary hemorrhage; however, its involvement in cardiovascular surgery is not well known. We present two cardiovascular surgical cases in which embolization of the hypertrophied bronchial artery was effective in controlling perioperative pulmonary hemorrhage.

**Case presentation:**

The first case was a 51-year-old man with chronic obstructive pulmonary disease who developed acute type A aortic dissection. After emergent surgery, his blood pressure suddenly dropped in the intensive care unit; computed tomography revealed a right hemothorax. Because a 4-mm dilated bronchial artery was identified on preoperative computed tomography, the hemothorax was suspected to be associated with bronchial artery hypertrophy. Selective bronchial arteriography was emergently performed and revealed a right pulmonary parenchymal blush. After subsequent coil embolization of the bronchial artery, the parenchymal blush disappeared, and his hemodynamic condition stabilized. The second case was a 66-year-old man with bronchiectasis who was referred for redo aortic valve replacement due to structural valve deterioration. A bioprosthesis was previously implanted to avoid permanent anticoagulation because the patient had repeated episodes of hemoptysis; however, he still had persistent hemosputum during admission for the redo aortic valve replacement. A dilated bronchial artery 3.7 mm in size was incidentally identified on preoperative computed tomography, and hence, the repeated hemosputum was suspected to be associated with bronchial artery hypertrophy. Bronchial arteriography revealed a right pulmonary parenchymal blush, and prophylactic embolization of the bronchial artery was performed. The hemosputum disappeared after the procedure, and redo aortic valve replacement was performed uneventfully 8 days later.

**Conclusion:**

In cardiovascular surgery, the risk of pulmonary hemorrhage associated with bronchial artery hypertrophy should be considered, especially in patients with inflammatory and ischemic respiratory diseases.

## Background

Pulmonary hemorrhage is a lethal complication of cardiovascular surgery. Unfortunately, its cause and prophylaxis are poorly understood. Inflammatory and ischemic respiratory diseases can cause the bronchial artery (BA) to become enlarged and frail, and subsequently prone to rupture [1]. This rare pathology, named bronchial artery hypertrophy (BAH), is associated with a potential risk of pulmonary hemorrhage. However, the involvement of BAH in perioperative pulmonary hemorrhage in cardiovascular surgery patients remains unclear. We present two cases in which BAH embolization was effective in controlling perioperative pulmonary hemorrhage in patients undergoing cardiovascular surgery.

## Case presentation

### Case 1

**Fig. 1:**
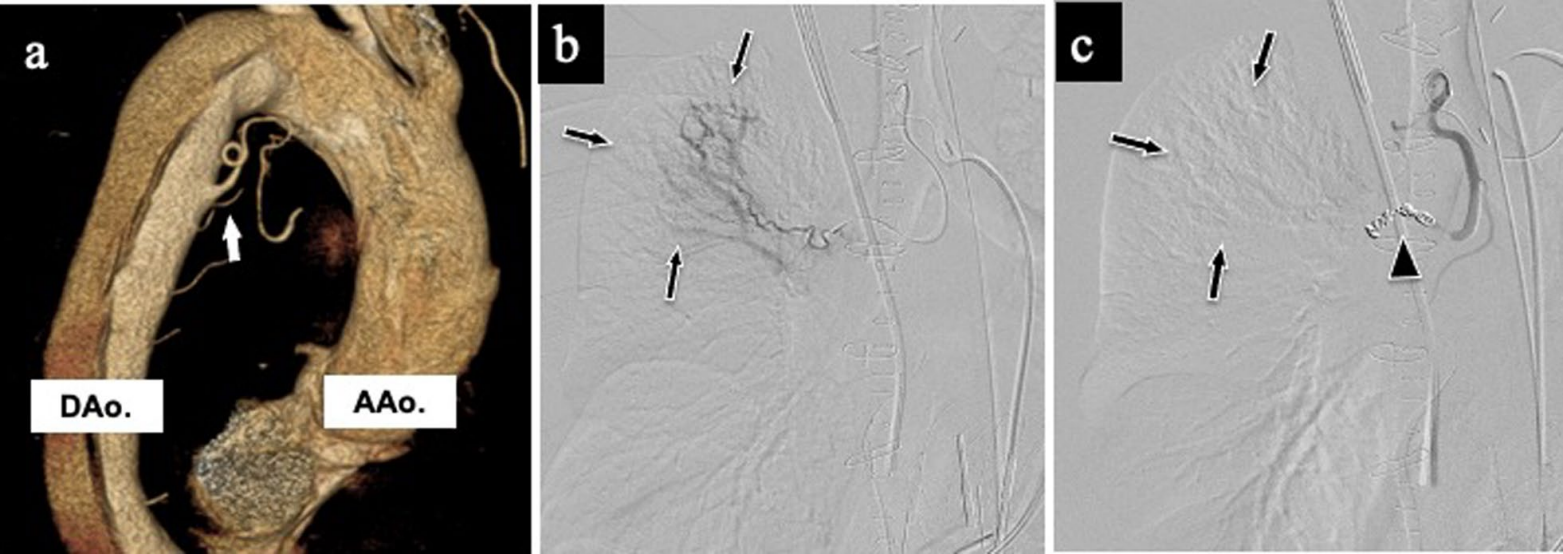
Preoperative computed tomography and postoperative angiography images in case 1: **a** BAH identified on preoperative three-dimensional CT image (white arrow); **b** pulmonary parenchymal blush in the right upper lobe demonstrated on BA angiography (arrows); **c** disappearance of parenchymal blush (arrows) after embolization (arrowhead) of the BA. *BAH* bronchial artery hypertrophy, *CT* computed tomography, *BA* bronchial artery, *AAo* ascending aorta, *DAo* descending aorta

A 51-year-old man with chronic obstructive pulmonary disease (COPD) complained of sudden chest pain and was transferred to our institution. Contrast-enhanced computed tomography (CT) revealed a type A aortic dissection, and he underwent emergency surgery successfully. However, after transfer to the intensive care unit, his systolic blood pressure suddenly dropped to 60 mmHg, and oxygen saturation decreased to < 90%. An emergency CT revealed massive right hemothorax; a chest tube was subsequently inserted, draining approximately 3000 ml of bloody fluid in the initial 2 h after insertion. The preoperative CT was retrospectively reviewed and a 4-mm dilated BA was identified. Pulmonary hemorrhage was suspected to be associated with BAH (Fig. 1a). We requested bailout surgery from a thoracic surgeon and also decided to perform an endovascular BA assessment while waiting for the surgeon’s arrival. Because emergent selective bronchial arteriography revealed a right pulmonary parenchymal blush (Fig. 1b), subsequent coil embolization was performed. After the embolization, the parenchymal blush disappeared, and his hemodynamic condition stabilized (Fig. 1c). He was extubated and discharged after 9 and 63 postoperative days, respectively.

### Case 2

**Fig. 2:**
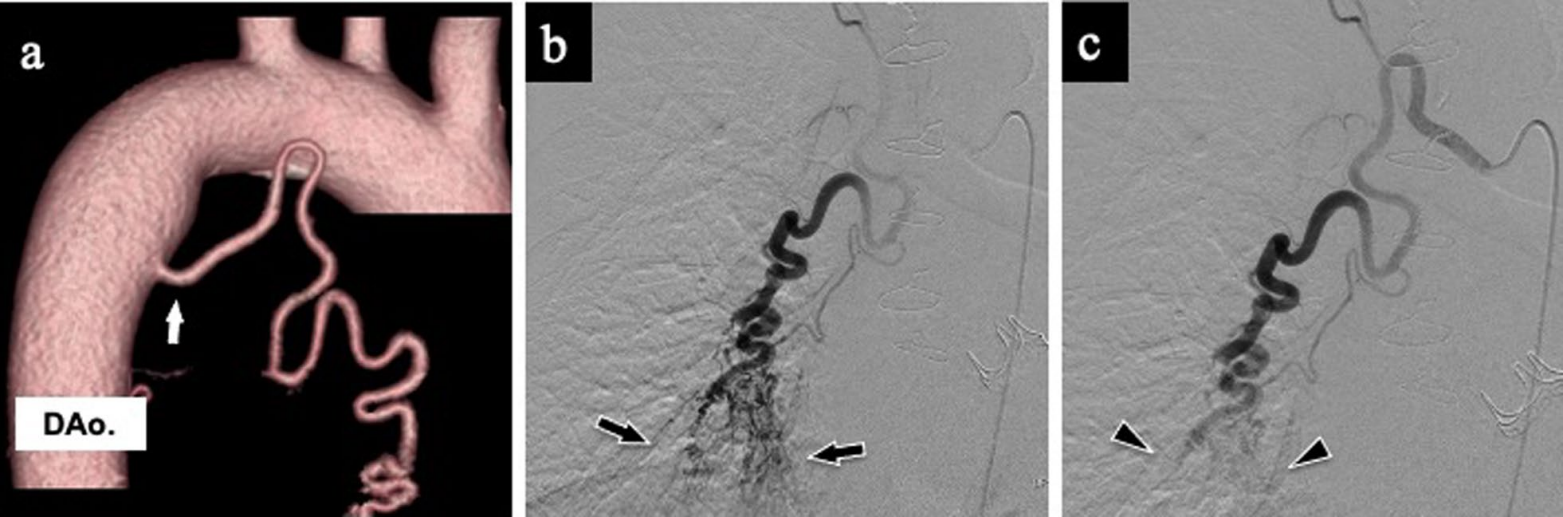
Preoperative computed tomography and angiography images in case 2: **a** BAH identified on preoperative three-dimensional CT image (white arrow); **b** pulmonary parenchymal blush in the right lower lobe demonstrated on BA angiography (arrows); **c** disappearance of parenchymal blush after embolization using a liquid embolic agent (arrowheads). *BAH* bronchial artery hypertrophy, *CT* computed tomography, *BA* bronchial artery, *DAo* descending aorta

A 66-year-old man with bronchiectasis was referred to our hospital for redo aortic valve replacement (AVR) due to structural valve deterioration. He was 48 years old at the initial surgery, but a bioprosthesis was implanted because of concerns regarding possible exacerbation of recurrent hemoptysis if permanent anticoagulation therapy would be required after mechanical valve implantation. He had persistent hemosputum even after AVR with a bioprosthesis. Before the redo AVR, a dilated BA with a size of 3.7 mm was incidentally identified on preoperative CT (Fig. 2a). Thus, we suspected that the recurrent hemoptysis was associated with BAH and could be exacerbated during the redo AVR. Therefore, prophylactic embolization of the BA was planned. Because bronchial arteriography revealed a right pulmonary parenchymal blush (Fig. 2b), BA embolization with a liquid embolic agent was performed, and the hemosputum resolved (Fig. 2c). Eight days after the procedure, a repeat AVR was performed uneventfully.

## Discussion

The BA is usually < 1.5 mm in diameter and is difficult to identify on routine CT. BAH is diagnosed when the BA is > 2 mm in diameter. Although the precise incidence of BAH remains unclear, various types of respiratory diseases, including inflammatory, infectious, and ischemic diseases, are known to be associated with BAH, and possible mechanisms have been proposed for each etiology. Increased neovascularity and microvascular thrombosis can promote the expansion of the BA in inflammatory and infectious diseases, such as bronchiectasis and tuberculosis [2, 3]. In contrast, tissue ischemia can cause compensatory BAH in chronic lung ischemia, such as in COPD and chronic thromboembolic pulmonary hypertension [2]. In these pathologies, normal vascular anastomoses between the bronchial and pulmonary arteries are more prominent and increase blood flow through the BAH [3, 4]. Furthermore, it has been pointed out that new collateral vessels promoted by infectious and inflammatory diseases have thin walls and are prone to rupture [1, 2]. There are, however, few reported cases of catastrophic pulmonary hemorrhage during cardiac surgery, specifically in patients diagnosed with BAH [5]. Potential mechanisms of pulmonary hemorrhage during cardiac surgery have been reported to be catheter-induced pulmonary artery perforation, airway bleeding caused by traumatic intubation, and comorbid respiratory diseases [6, 7]. In our cases, BAH was highly suspected to be associated with perioperative pulmonary hemorrhage because the pulmonary parenchymal blush supplied by the BA was identified on angiography, and the bleeding subsided after BA embolization. Furthermore, embolization is effective not only for the treatment of ongoing bleeding, but also for prophylaxis of possible exacerbation of hemoptysis. Although our cases had no remarkable complications following BA embolization, there are some reports of complications such as transverse myelitis and bronchial infarction after BA embolization [2]. Therefore, it should be applied only in situations where BAH is highly suspected to be associated with a perioperative hemorrhage. Additionally, because selective BA cannulation is a technically demanding procedure, surgical repair including lobectomy should always be considered in case of a massive hemothorax [8, 9]. The diagnosis of BAH and the consideration of therapeutic and prophylactic embolization of the BA could reduce the risk of mortality due to lethal perioperative pulmonary hemorrhage, especially in patients with inflammatory, infectious, and ischemic respiratory diseases.

## Conclusion

BAH may be associated with perioperative pulmonary hemorrhage in cardiovascular surgical patients. Preoperative assessment of the bronchial arteries could help reduce the potential risk of perioperative pulmonary hemorrhage, especially in patients with inflammatory, infectious, or ischemic respiratory diseases. When BAH is suspected to be associated with perioperative pulmonary hemorrhage, bronchial arteriography and embolization may be desirable.

## Data Availability

The data that support the findings of this study are available from the corresponding author upon reasonable request.

## Abbreviations

AVR: Aortic valve replacement
BA: Bronchial artery
BAH: Bronchial artery hypertrophy
COPD: Chronic obstructive pulmonary disease
CT: Computed tomography
